# Interfaces Between Cathode and Electrolyte in Solid State Lithium Batteries: Challenges and Perspectives

**DOI:** 10.3389/fchem.2018.00616

**Published:** 2018-12-12

**Authors:** Kaihui Nie, Yanshuai Hong, Jiliang Qiu, Qinghao Li, Xiqian Yu, Hong Li, Liquan Chen

**Affiliations:** ^1^Renewable Energy Laboratory, Institute of Physics, Chinese Academy of Sciences, Beijing, China; ^2^University of Chinese Academy of Sciences, Beijing, China

**Keywords:** cathode, solid electrolyte, solid state lithium battery, cathode-solid electrolyte interface, advanced characterization

## Abstract

Solid state lithium batteries are widely accepted as promising candidates for next generation of various energy storage devices with the probability to realize improved energy density and superior safety performances. However, the interface between electrode and solid electrolyte remain a key issue that hinders practical development of solid state lithium batteries. In this review, we specifically focus on the interface between solid electrolytes and prevailing cathodes. The basic principles of interface layer formation are summarized and three kinds of interface layers can be categorized. For typical solid state lithium batteries, a most common and daunting challenge is to achieve and sustain intimate solid-solid contact. Meanwhile, different specific issues occur on various types of solid electrolytes, depending on the intrinsic properties of adjacent solid components. Our discussion mostly involves following electrolytes, including solid polymer electrolyte, inorganic solid oxide and sulfide electrolytes as well as composite electrolytes. The effective strategies to overcome the interface instabilities are also summarized. In order to clarify interfacial behaviors fundamentally, advanced characterization techniques with time, and atomic-scale resolution are required to gain more insights from different perspectives. And recent progresses achieved from advanced characterization are also reviewed here. We highlight that the cooperative characterization of diverse advanced characterization techniques is necessary to gain the final clarification of interface behavior, and stress that the combination of diverse interfacial modification strategies is required to build up decent cathode-electrolyte interface for superior solid state lithium batteries.

## Introduction

The daily increasing energy consumption demands advanced batteries with higher energy density and superior safety performance, particularly for large-scale applications like electric vehicles and grid storage (Tarascon and Armand, 2001). In solid state lithium batteries, conventional liquid electrolyte based on flammable carbonate components is replaced by solid electrolyte. Thereby, the safety concern related to thermal runaway and electrolyte combustion is likely to be much mitigated (Zhang et al., 2013). Owing to the mechanical properties of solid electrolyte, solid state lithium batteries could resist lithium dendrite in a great degree and the cycle life could be extended longer than lithium batteries based on liquid electrolyte. The wide electrochemical stability window of solid electrolyte may further enable the application of Li metal as anode and cathodes with even higher oxidization potential. With larger lithium chemical potential difference between anode and cathode, the energy storage can be much improved correspondingly. Owing to these glittering properties, solid state lithium batteries have attracted much research attention in recent years and become promising candidates for next generation energy storage devices with the expectations of improved safety performance, longer cycle life, and higher energy density (Bates et al., 2000; Duan et al., 2017). Depending on whether the battery contains liquid electrolyte or not, solid state lithium batteries can be divided into all solid state lithium batteries and hybrid solid liquid electrolyte lithium batteries (Cao et al., 2018).

The development of practically accessible solid state lithium batteries is hindered by two major bottle-necks. The first one is the low ionic conductivity of solid electrolyte, which is several-orders-lower than that of liquid electrolyte at room temperature (RT). Continuous research efforts have been devoted to designing superior solid electrolyte in the past decades, and much progress has been achieved so far. Up to now, the RT ionic conductivities of some systems have approached or even surpassed that of liquid electrolytes. RT conductivities of NASICON-type oxides (Aono et al., 1990; Fergus, 2012) and lithium garnets (Murugan et al., 2007) have reached ~1 mS cm^−1^. Kato et al. further increased the number to ~25 mS cm^−1^ in Li_9.54_Si_1.74_P_1.44_S_11.7_Cl_0.3_ (Kato et al., 2016). Decent solid electrolytes are now available with intrinsically high Li^+^ conductivity, Lithium ion transference number t_Li+_ ~1, and particularly no desolvation step compared to organic liquid electrolytes, t_Li+_ is around 0.2 0.5 (Xu, 2004; Zugmann et al., 2011). Corresponding solid state lithium batteries are expected to exhibit large capacities and high power densities for future applications (Kato et al., 2016).

Despite the rapid development of solid electrolyte itself, the even more serious hinderance for solid state lithium batteries is the high interfacial resistance caused by poor contact and interfacial reactions (Zhang et al., 2016). Without liquid fluidity, it's challenging to obtain intimate contact between solid electrolyte and electrode. The periodic electrode expanding and shrinking during cycle further deteriorates the mechanical particle-to-particle contact. As a consequence, high polarization, and low utilization of active materials are conventional in solid state lithium batteries. Meanwhile, the high voltage instability of solid electrolyte is another noteworthy concern for solid state lithium batteries. Solid electrolytes are expected to provide wider electrochemical stability window compared with liquid electrolyte. Literatures also reported wide electrochemical window up to 5.0 V or even 6.0 V in inert electrode system. However, some computation results and experimental results confirmed that the window is not as high as reported before (Han et al., 2016). Especially, for SPE the prevailing experimental reports of which are mostly based on LiFePO_4_ cathode cycled within 3.8 V. Considering the catalytic behavior of transition metal oxides, the practical stability of solid electrolyte at high voltage still needs further investigation, and verification. Moreover, the electric potential profile across the electrode-electrolyte interface is still a problem unanswered, which has significant influences on interface reaction and battery performance. Many investigations have been carried out on the abrupt change of electric potential across cathode-electrolyte interface (Liang et al., 2018). And it has been pointed out that the interfacial side reactions may be accelerated dramatically due to the specific local electric potential. However, an intensive and systematic understanding is still lacking on the potential profile distribution across the interface and its corresponding influence on the interface behavior.

It can be inferred from above that the key to realize solid state lithium batteries with competitive performance mostly relies on the construction of a stable and intimate interface, where different strategies have been developed. Direct co-sintering of electrode and electrolyte may be an effective and simple method to achieve good interfacial contact. However, the high temperature facilitates ion interdiffusion across interface, leading to side reactions between the electrode and solid electrolyte. *In-situ* synthesis of solid electrolyte or cathode is another promising choice, but necessary sintering procedure also encounters the problem of ion interdiffusion. Due to the interfacial passivation layer formation, the dynamics performance of solid state lithium batteries may be deteriorated. Diverse strategies have been proposed to build up proper artificial interlayer, including cathode coating, interface softening, buffer layer introducing, and etc. These strategies can effectively improve the physical contact, diminish interfacial side reactions, and mitigate the space charge layer (SCL) in sulfide solid electrolyte, but corresponding solid state lithium batteries are still far from practical applications. Till now much efforts have been devoted to interface modification and progresses have been obtained, but interface property is still a major obstacle on the way to practical solid state lithium batteries.

Interface research has become a challenging but hot topic in solid state batteries (Gao et al., 2018) (Lu et al., 2018; Xu et al., 2018). The interface between lithium anode and solid electrolyte has been extensively investigated. Note that the cathode-solid electrolyte interface serves as a hinge to obtain batteries with improved safety, longer cycle life, and higher energy density. So, the interface between cathode and solid electrolyte is equally important to the interface at anode side. Here in this review, we put a special focus on the fundamental issues about cathode-solid electrolyte interfaces in solid state lithium batteries based on diverse cathode-electrolyte materials. We hope to summarize the previous understandings and recent advances on the interface research. Furthermore, we hope to shed light on the possible approach to the final understanding of interface phenomenon with advanced characterization techniques. In chapter 2, we present a brief overview on basic principle of battery operation and scientific issues relevant to interface layer. In chapter 3, the interfacial problems between cathodes and four kinds of prevailing solid electrolytes are specifically discussed, corresponding optimization methods are also introduced. In chapter 4, advanced characterization techniques used for the investigation of solid-solid interface behavior are consolidated, corresponding advances and achievements are summarized. Finally, we give a comprehensive conclusion about the cathode-solid electrolyte issues and perspectives for building favorable interfaces.

## Basic Principle and Issues at the Cathode-Solid Electrolyte Interface

Solid state lithium batteries have three major components cathode, anode, and solid electrolyte. The cathode material herein refers to the same lithium-containing compound as the lithium ion battery. During charging, Li^+^ are extracted from the cathode and migrate to anode via solid electrolyte, while electrons transfer from the cathode to anode through external circuit. In this process, oxidation and reduction reactions take place at the cathode and anode sides, respectively. During discharging, Li^+^ and electrons migrate toward the reverse direction, accompanied with cathode reduction, and anode oxidation. The following reaction steps are involved at electrode-electrolyte interface in solid state lithium batteries: (i) Li^+^ diffusion in the electrolyte, (ii) Li^+^ hop into the first lattice site of the electrode and oxidation/reduction reaction happened at the same time i.e., the charge transfer process, (iii) Li^+^ diffusion in the electrode, and (iv) Surface reaction, etc. A stable and intimate interface is necessary to ensure the above reaction steps proceed smoothly.

**Figure 1:**
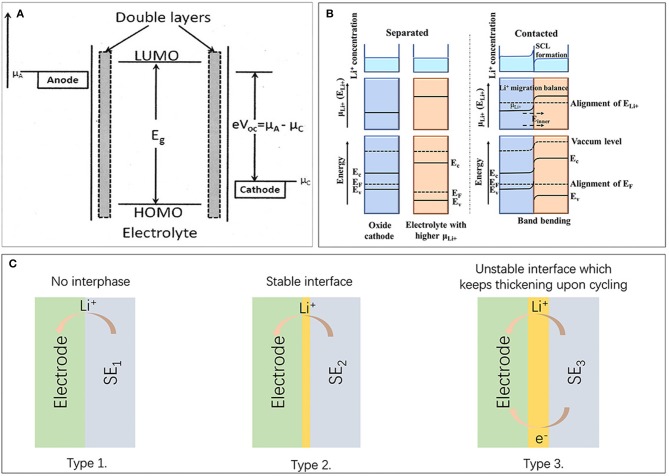
**(A)** Relative energies of μ_A_ and μ_C_ vs. the LUMO-HOMO window of the electrolyte. [Reprinted with permission from Goodenough (2013). Copyright (2013) American Chemical Society]. **(B)** Illustration of ionic and electronic structures of electrode and electrolyte before (left) and after contact (right). Shown is a mixed ionic and electronic conducting electrode in contact with a pure ionic conducting solid electrolyte with higher Li^+^ chemical potential. **(C)** Illustration of three possible types of the solid electrolyte/solid electrode interfaces.

Interface instability may derive from chemical or electrochemical problems, a most fundamental origin is the abrupt electrochemical potential change at electrode-electrolyte interface. As illustrated in Figure 1A, the lowest unoccupied molecular orbital (LUMO) and highest occupied molecular orbital (HOMO) of electrolyte determines the electrochemical stability window of solid state lithium batteries. The electrochemical potential of anode and cathode is marked as μ_A_ and μ_C_, which need to match with the electrochemical window of electrolyte to achieve thermodynamic stability (Goodenough, 2013). Figure 1B shows the ionic and electronic structures of electrode and electrolyte before and after contact, where electrolyte exhibits higher Li^+^ chemical potential. Li^+^ will migrate from solid electrolyte to oxide cathode to achieve thermodynamic equilibrium. This will lead to the alignment of Li^+^ electrochemical potentials and a space charge layer (SCL) formation with an inner electric field after contacted. While band bending and alignment of Fermi level will happen due to the formation of a heterojunction. As a result, original position of energy levels, inner electric field formation, band bending as well as the energy levels change during charging/discharging determine barriers for charge carriers transfer. From the barriers for charge carriers transfer, whether electrons/holes could transfer at the interface i.e., the oxidation/reduction of the electrolyte, could be concluded. (Hausbrand et al., 2014) It was pointed out that side reactions at interface may be further accelerated due to the large polarization from electric potential (φ) drop (Ohta et al., 2006; Zhou et al., 2016). In such cases, solid electrolyte decomposition and intermediate transition layer formation may take place at interface. In addition, conventional high temperature processing may further induce interfacial interdiffusion of TM (transition metal) elements and favor the formation of specific transition region.

Based on the intrinsic properties of different kinds of solid electrolytes and cathode materials, there are mostly three types of electrode-electrolyte interfaces in solid state lithium batteries, as shown in Figure 1C (Zhu et al., 2016). Type 1 is a stable interface scenario with no electrolyte decomposition or chemical side reactions. This is the ideal interface, which seldomly appears in practical systems. Type 2 represents interface which is electronic insulating but provides Li^+^ migration channels. Within this scheme, further interfacial side reactions can be suppressed and battery operation can be maintained. The LiCoO_2_/LiPON interface may be a proper example for this case (Zhu et al., 2016). Type 3 is an undesirable but most common interface with mixed ionic and electronic conductivity. In this scheme, continuous side reactions occur, and battery fade happens, as in LiCoO_2_/LGPS interface. Depending on the intrinsic property of electrode and electrolyte, different types of interfaces will be built up, but only type 1 and 2 are accessible for practical applications. By introducing proper buffer layers between cathode and electrolyte, a stable artificial layer can be constructed and convert interface from type 3 to type 2. Considering the significance of building proper Li^+^ conducting layer and balancing interfacial potential drop, we will present detailed discussion in following chapters according to the characteristics of specific solid electrolytes.

**Figure 2:**
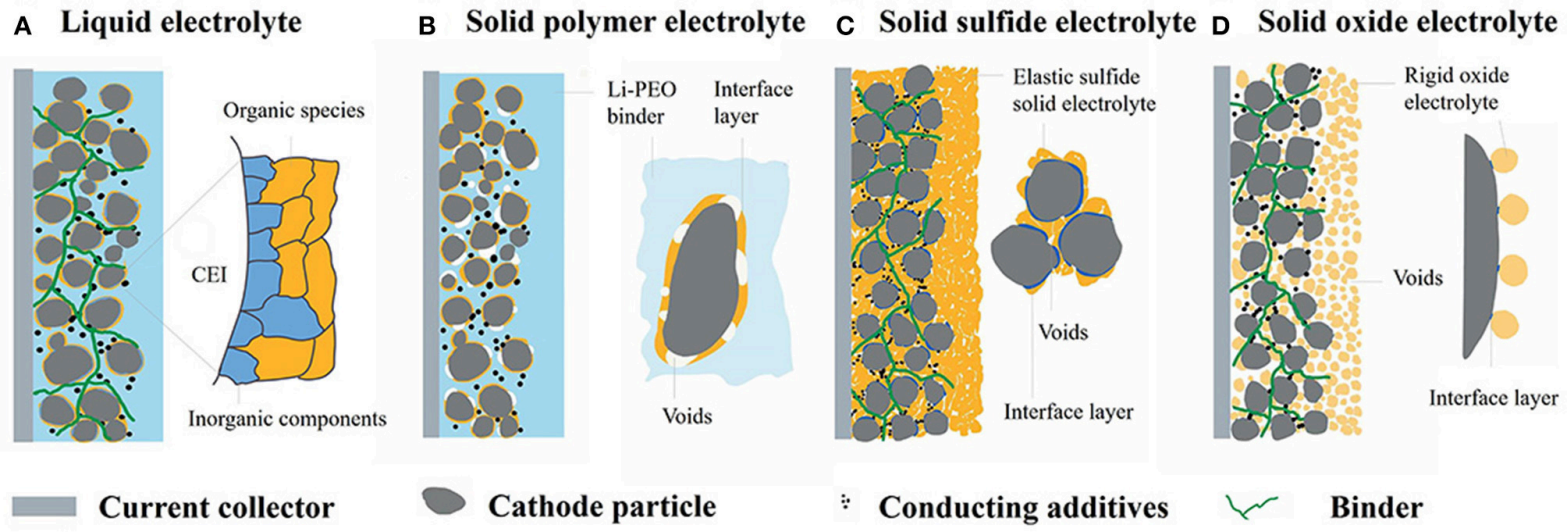
Models of morphology at the interface between cathode-electrolyte: **(A)** The cathode particles are totally immersed in a liquid electrolyte and an interface layer will form. **(B)** Cathode particles are distributed in a Li-PEO binder with good contact while voids will generate upon cycling because of the interfacial reactions or pulverization of cathode particles. **(C)** Sulfide particles have favorable mechanical properties as ductility and deformability, which could change its shape to match with the rigid solid electrode. **(D)** Solid oxide electrolyte: Poor point-contact will form due to the rigid ceramic nature. Interface layer will form in all the aforementioned system if decomposition reactions or interdiffusion occurred at the interface.

Apart from the chemical stability of the interface, mechanical behavior also has a significant impact on battery performance. In conventional lithium ion batteries based on liquid electrolyte, cathode particles can be totally immersed in liquid electrolyte and passivation layer called solid electrolyte interphase (SEI) may form. Good contact between electrode and liquid electrolyte could therefore be maintained throughout battery cycle, Figure 2A (Liu et al., 2006; Takamatsu et al., 2012). However, it is challenging to maintain intimate electrode-electrolyte interface in solid state lithium batteries, especially over many cycles (Goodenough, 2013). The deficient contact in solid state lithium batteries may well-lead to low utilization of active particles, large polarization and even contact loss during cycle.

Due to the distinguished mechanical properties, there is distinct difference in contact behavior among various types of electrolytes. Solid electrolytes can be generally classified into SPE and solid inorganic electrolyte, the latter can be further classified into solid oxide and solid sulfide electrolyte. Polymer electrolyte has moderate contact with cathode due to the elasticity and deformability of organic polymers. Nevertheless, vacant cavities will still generate due to interface reactions and cathode pulverization during cycling (Figure 2B) (Nakayama et al., 2010). The effective contact area between cathode and polymer electrolyte will consequently reduce with battery cycle. Due to reasonable mechanical ductility, deformable sulfide particles could also change its shape to match with cathode particles. Hence, the poor contact between electrode and sulfide electrolyte can be much improved by mechanical pressing (Figure 2C) (Sakuda et al., 2013; Ito et al., 2017). While contact loss will also happen upon cycling along with the shrinkage and expansion of cathode particles (Koerver et al., 2017). Solid oxide electrolytes have the worst point-contact with cathode due to the rigid ceramic nature (Figure 2D) (Ohta et al., 2013, 2014). The insufficient mechanical contact facilitates cathode particles completely isolated from solid electrolyte, i e., the “dead” area. Due to the lack of percolation paths, neither electrons nor Li^+^ can be transferred from/into the dead areas. The “dead” areas not only lead to direct capacity fading, but also induce locally strong non-uniform current and strain distribution (Zhang et al., 2018). The poor solid-solid contact typically brings about large polarization and low capacity. To improve the interface contact, various strategies have been adopted, such as *in-situ* synthesis of solid electrolyte, interface buffer layer, cathode coating, gel system etc. Based on different properties of various electrolytes, specific strategies will be adopted, and introduced specifically in Chapter 3.

## Challenges and Solutions on Interfaces Between Cathode and Diverse Solid Electrolytes

### Interface Between Cathode and Solid Polymer Electrolyte

After Wright's discovery of alkali metal ions conductivity in poly(ethylene oxide) (PEO) in 1973 (Fenton et al., 1973), Armand firstly proposed PEO with lithium salt as solid electrolyte for solid state lithium batteries (Armand, 1983). PEO-based SPE is widely accepted as a most promising candidate for solid state lithium batteries owning to its advantages such as easy fabrication, low cost and excellent compatibility with lithium salt. In SPE, Li^+^ can migrate in the free volume of polymer host assisted by the motion of the polymer chains when temperature is above T_g_ (glass transition temperature) (Bruce, 1995). In PEO based SPE, Li^+^ were coordinated by ether oxygen and transport with the breaking/forming of Li-O bonds (Bruce, 1995; Xu, 2004). However, PEO-based SPE is not stable above 4.0 V, which confines the pairing cathode operating within low-voltage range. In most literature reports, the prevailing choice is LiFePO_4_ (Croce et al., 2001). Considering the catalytic effect of transition metal oxides, PEO decomposition may well-be triggered at the interface region. And improving the antioxidant properties of SPE to high-voltage range is essential to realize high energy density solid state lithium batteries based on PEO (Fan et al., 2002). There are mostly three types of SPE available (i) dry solid-state polymer, (ii) gel/plasticized polymer electrolyte, and (iii) polymer composites (Manuel Stephan, 2006). Diverse optimization strategies have been utilized for different SPE systems, as discussed below.

**Figure 3:**
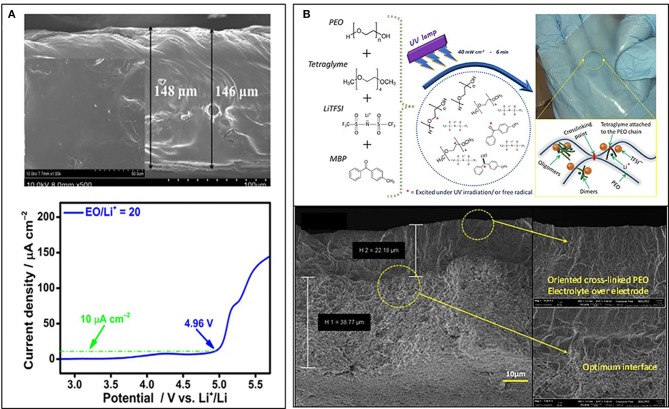
**(A)** SEM image (up) and linear sweep voltammogram (down) for the membrane of the LiTNFSI/PEO (EO/Li^+^ = 20) blended polymer electrolyte. [Reprinted with permission from (Ma et al., 2016b). Copyright (2016) American Chemical Society]. **(B)** Interconnected PEO chains with hypothesized branched clusters of tetraglyme oligomers (top left) and the real aspect of a freshly prepared ISPE (top right); Cross-sectional FESEM images showing the optimum interface achieved after UV curing (down). [Reprinted with permission from (Porcarelli et al., 2016). Copyright (2016) Nature].

“Dry” solid-state polymer refers to PEO, PPO, PAN etc. and their derivatives containing Li salt, corresponding optimization has been focused on polymer architecture and Li salt selection. On the electrochemical instability of PEO-based SPE above 4.0 V (Croce et al., 2001), Nakayama et al. (2010) proposed a model that two sequential factors affect cyclic degradation. The first one is the local current enhancement induced by cathode pulverization, which can be attributed to the solid-solid contact between electrolyte and electrode. The second is continuous and uneven decomposition of TFSI [N (CF_3_SO_2_)${}_{2}^{-}$] due to local polarization. These results indicate that the mechanical property of SPE and the Li salt selection are both essential. Ma et al. proposed a novel SPE composed of PEO and extra-stable lithium salt- (Li[(CF_3_SO_2_)(n-C_4_F_9_SO_2_)-N], LiTNFSI). This SPE exhibits a homogeneous and compact morphology and high electrochemical stability at ~4.0 V vs. Li^+^/Li (Figure 3A). Long-term cycling stability and sufficient thermal stability (>350°C) was also obtained in this novel SPE (Ma et al., 2016b).

Improving the antioxidative capability of PEO is another critical aspect to promote high voltage interface stability. Copolymerization, branching, and crosslinking are common polymer modification methods, which also favor designing more antioxidative polymers (Tong et al., 2014; Porcarelli et al., 2016; Wang et al., 2016a). UV-induced (co)polymerization can promote effective interlinking between polyethylene oxide (PEO) chains plasticized by tetraglyme. Hereby, Porcarelli et al. (2016) obtained SPE with wide electrochemical stability window (>5 V vs. Li/Li^+^) from LSV test. Figure 3B illustrates the synthesis process and photograph of as-prepared SPE, together with cross-sectional FESEM images of optimized cathode-electrolyte interface. It is clear that electrolyte creates conformal coating by following the contours of active particles, which leads to improved active materials utilization. Similar optimized SPE have also been achieved by a PEO and liquid-crystalline copolymers with small molecular liquid crystals as fillers. High ionic conductivity, lithium ion transference number combined with wide electro-chemical stability window of the copolymer facilitated a good electrochemical performance (Tong et al., 2014).

Except for developing PEO derivatives, exploiting other antioxidative polymer electrolyte has also attracted much attention (Zhang et al., 2015; Chai et al., 2017). Chai et al. (2017) prepared a kind of novel poly (vinylene carbonate) (PVCA)-based SPE which possessed both interfacial compatibility with Li anode and high-voltage LiCoO_2_ cathode (4.3 V vs. Li/Li^+^). From *in-situ* polymerization of PVCA, polymer electrolyte can be even incorporated into the porous cathodes and the effective contact area can be much increased as a result. Owing to the good contact and compatibility, the battery exhibited high discharge capacity and excellent cycling performance.

The second category SPE is called “gel polymer electrolyte” or “plasticized polymer electrolyte” which contains both liquid and solid components. Thus, gel polymers possess both cohesive properties of solids and the diffusive property of liquids (Manuel Stephan, 2006) which makes hybrid solid liquid electrolyte lithium batteries have unique advantages (Huang et al., 1996). As reported, by modifying PEO electrolyte with plasticizing liquid dimethyl sulphoxide (DMSO) (< 5%), electrochemical stability window can be extended above 4.1 V, exceeding Fermi levels of several high voltage cathodes. The long terms cycling stability was also improved obviously (Zewde et al., 2018). By phase inversion technique, Deng et al. prepared a microporous polymer electrolyte (MPE) based on poly (vinylidene fluoride) (PVDF)/PEO star polymer, which exhibits wide electrochemical stability window of ~5V (Deng et al., 2015).

Solid composite electrolyte, as SPEs subset, is another competitive candidate among kinds of SPEs. Solid composite electrolyte combined the virtues of both polymer and ceramic, exhibiting excellent mechanical stability, high ionic conductivity, wide electrochemical stability window, intimate contact performance, and etc. Relevant researches will be specifically introduced in Chapter 3.4.

Apart from solid electrolyte modification, cathode surface modification is another effective way to mitigate the interface degradation. Note that a principle factor that restricts PEO application at high voltage is the strong oxidation/catalytic property of TM oxides cathode. Consequently, surface modification on cathode material becomes another way to enable PEO operation at high voltage. Yang et al. synthesized continuous and compact LATP coating layer on LiCoO_2_ through solution-procession and low temperature treatment. solid state lithium batteries assembled with PEO-based SPE and LATP modified LiCoO_2_ shows high capacity retention (93.2% after 50 cycles) at 4.2 V, which suggests that surface coating can effectively suppress PEO oxidation at high voltage (Yang et al., 2018). By cathode coating, PVCA-coated LiCoO_2_ also showed much enhanced cycling stability of PEO based SPE at 4.45V (Ma et al., 2017).

### Interface Between Cathode and Solid Oxide Electrolyte

Oxide-based solid electrolytes exhibit good chemical stability against air and compatibility with high voltage cathodes. Typical solid oxide electrolytes include garnet-type Li_7_La_3_Zr_2_O_12_ (LLZO), NASICON-type LiTi_2_(PO_4_)_3_, LiSICON-type Li_14_Zn (GeO_4_)_4_, and Perovskite-type La_0.5_Li_0.5−δ_TiO_3_ (LLTO). Solid oxide electrolyte is a most competitive choice for solid state lithium batteries (Chen et al., 1980; Delmas et al., 1988; Inaguma et al., 1993). However, there are two major challenges for solid oxide electrolytes. The first one is the generally low ionic conductivity, which is lower than sulfide electrolytes. Despite the phenomenally low intrinsic bulk conductivity, recent investigations point to the high interface polarization that restrains battery dynamics. The second challenge is the rigid ceramic nature, which causes poor point-contact at electrode-electrolyte interface, as discussed above. Solid oxide electrolytes have a key advantage of intrinsic wide electrochemical window. For garnet-type electrolytes, the experimental value can be even wide as ~0–6 V (Li et al., 2015; Thangadurai et al., 2015). Among all solid oxide electrolytes, garnet-type electrolyte is an attractive candidate due to its high RT ionic conductivity (~1 mS cm^−1^), practically wide electrochemical window and chemical stability against Li etc. In the following discussion, we mostly take garnet LLZO as a typical example to discuss the interfacial problems solid oxide electrolyte faced with, other systems are briefly mentioned at the end.

Since interfacial resistance from poor contact (Figure 2D) is proven to be the main reason for the high internal resistance of solid state lithium batteries (Park et al., 2016; Han et al., 2017), quite a few approaches have been applied to reduce interfacial resistance, including co-sintering (Wakayama et al., 2016), *in-situ* synthesized electrolyte layer (Yoshima et al., 2016; Kazyak et al., 2017), interface buffer layers (Kato et al., 2014; Park et al., 2016), interface softening (Seino et al., 2011; Sakuda et al., 2012; Liu et al., 2016), surface coating (Han et al., 2017), and amorphous cathode (Matsuyama et al., 2016; Nagao et al., 2017) etc.

**Figure 4:**
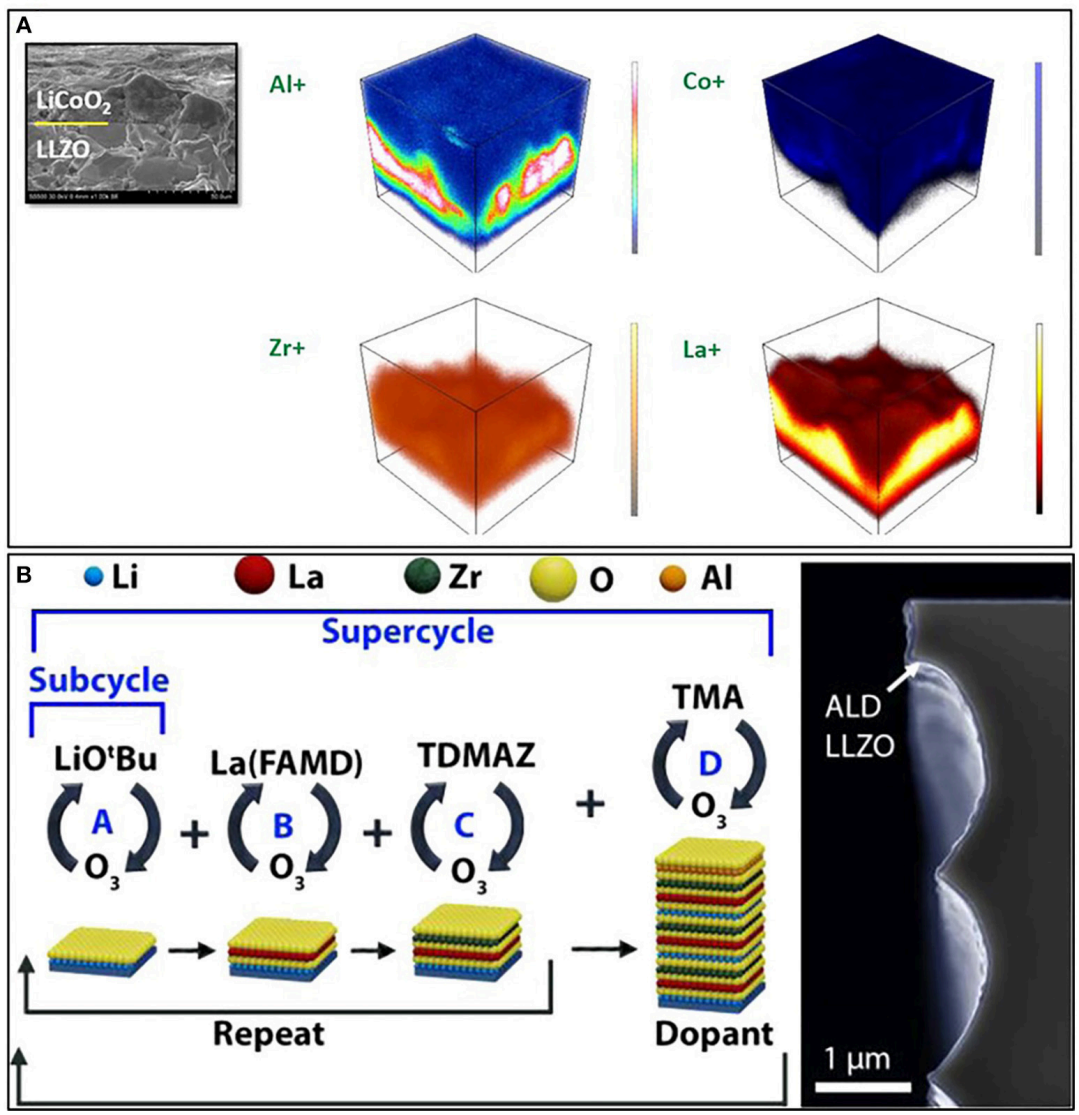
**(A)** TOF-SIMS-enabled three-dimensional elemental maps of the LiCoO_2_/ Li_7_La_3_Zr_2_O_12_ (LLZO) interface that is displayed in the inset SEM image. [Reprinted with permission from (Park et al., 2016). Copyright (2016) American Chemical Society]. **(B)** Schematic representation of an ALD super cycle composed of constituent binary ALD processes (left) and SEM image showing as-deposited ALD LLZO film (right). [Reprinted with permission from (Kazyak et al., 2017). Copyright (2017) American Chemical Society].

Electrode-electrolyte co-sintering, cathode layer *in-situ* synthesizing, and thin film deposition are proven effective in promoting surface contact, however necessary high-temperature handling (>500°C) will lead to elements interdiffusion, electrolyte decomposition and deteriorated performance (Wakayama et al., 2016). Tremendous efforts have been devoted to lower the sintering temperature of solid oxide electrolytes to mitigate the interdiffusion problem, while very finite progress has been achieved so far. With a combination of ab initio calculations, thermal analysis, and X-ray-diffraction, Ceder' group elucidated the decomposition reactions between high-voltage spinel cathode (Li_2_NiMn_3_O_8_, Li_2_FeMn_3_O_8_, LiCoMnO_4_) and solid oxide electrolyte (LLZO, LATP) (Miara et al., 2016). XRD revealed that spinel cathode and LLZO were not compatible with each other at 600°C, and the decomposition products can be predicted from calculated phase diagrams. In 2016, Park et al. (2016) studied the three-dimensional elemental distribution at LiCoO_2_/LLZO interface by TOF-SIMS. As illustrated in Figure 4A, Co diffuses into LLZO, and Zr/La diffuses into LiCoO_2_. While Al was leached out of LLZO, and diffuses into LiCoO_2_, cubic LLZO at the interface transformed to tetragonal phase. It was further proved that interface modification with Li_3_BO_3_ can reduce chemical cross-contamination and improve physical bonding.

Introducing interfacial buffer layer, such as Nb, LiNbO_2_, BaTiO_3_ was found to be an effective way to mitigate interface interdiffusion (Kato et al., 2014). By radio frequency (RF) magnetron sputtering, Kato et al. (2014) introduced a thin Nb layer (10 nm) on LLZO and then LiCoO_2_ was deposited on the Nb-modified LLZO by PLD at 600°C. *In-situ* synthesis of LiCoO_2_ by PLD guaranteed an intimate contact between cathode and solid electrolyte, while introducing Nb layer improved the interface performance by forming Li-Nb-O amorphous region. The Li^+^- conductivity of the amorphous Li-Nb-O region is high as 1 × 10^−6^ S cm^−1^, which will facilitate Li^+^ transport at interface (Glass et al., 1978). As a result, the mutual diffusion thickness is 40 nm, which is much smaller than the 100 nm Li^+^ insulating La_2_CoO_4_ region without Nb modification (Kim et al., 2011). Kazyak et al. presented a thermal ALD (atomic layer deposition) process which can significantly lower the formation temperature of the cubic phase to 555°C. The schematic processes and SEM images of LLZO products are illustrated in Figure 4B. Low melting compounds were also employed for good interfacial contact in solid state lithium batteries. Liu et al. used Li_3_BO_3_ (melting point ca. 700°C, Li^+^ conductivity ca. 2 × 10^−6^ S cm^−1^) as bonding aid for LCO/LLZO interface. Corresponding interface resistance reduced dramatically and electrochemical performance improved significantly. Yoshima et al. (2016) designed a gel to soften interface and achieved intimate contact at interface. LLZO coated with polyacrylonitrile (PAN)-based gel was prepared as electrolyte sheet, which reduced internal resistance of the whole battery. The assembled solid state lithium batteries exhibited good rate capability and cycling stability between −40 and 80°C. Very recently, Han et al. reported an all-ceramic cathode-electrolyte by thermally soldering LiCoO_2_ and LLZO together with Li_2.3−x_C_0.7+x_B_0.3−x_O_3_ solid electrolyte interphase can be spontaneously coated on both LLZO and LiCoO_2_ (Han et al., 2018). The simultaneous improvements in interfacial contact, (electro) chemical stability, ionic conductivity, and mechanical property of the all-ceramic cathode-electrolyte enabled an all solid state Li/LLZO/LiCoO_2_ battery with extremely high electrochemical performance.

Owing to the rigid ceramic nature, most solid oxide electrolytes face similar interfacial challenges when paired with solid cathode. The aforementioned interface modifying strategies can also be applied to diverse solid oxides electrolytes, except that typical solid oxide electrolyte are further hindered by other factors. Perovskite-type LLTO was firstly prepared by Inaguma et al. (1993) which exhibits low ionic conductivity across grain boundary (around 10^−5^ S cm^−1^) and poor stability with anode (instable below 1.8 V vs. Li^+^/Li). As a result, most works on LLTO focus on improving ionic conductivity and chemical stability vs. Li anode (Chen and Amine, 2001; Kotobuki et al., 2011; Huang et al., 2016). Li_1+x_Al_x_Ge_2−x_(PO_4_)_3_ (LAGP) and Li_1+x_Al_x_Ti_2−x_(PO_4_)_3_ (LATP) are two common NASICON-type solid oxide electrolyte. Because of Ti^4+^ reduction, LATP suffers redox reaction at 2.5 V vs. Li^+^/Li. Although LATP shows high ionic conductivity (Delmas et al., 1988), its incompatible with low potential anodes, especially Li, confines its application in solid state lithium batteries. In these solid oxide electrolytes related research, interface softening, and *in-situ* synthesizing have also been carried out (Kim et al., 2017; Zhang et al., 2017b) and corresponding investigations are still in progress.

### Interface Between Cathode and Solid Sulfide Electrolyte

Solid sulfide electrolytes are the derivatives of solid oxide electrolytes by substituting oxygen with sulfur. Since the electronegativity of S is less than O, Li^+^ binding energy is smaller and Li^+^ can move more freely. Among all solid electrolytes, solid sulfide electrolyte exhibits the highest Li^+^ conductivity. Another attractive feature of solid sulfide electrolyte is their mechanical property. These materials exhibit plastic deformation under mechanical pressure, and this softness makes it possible to prepare densely packed interface (Koerver et al., 2017). In recent years, the research focus of solid sulfide electrolyte is Li_2_S-P_2_S_5_ based systems, which exhibit superior Li^+^ conductivity, electrochemical stability, and mechanical properties. According to the composition difference, Li_2_S-P_2_S_5_ system can be divided into binary solid sulfide electrolyte (composed of Li_2_S and P_2_S_5_, such as Li_3_PS_4_, Li_7_P_3_S_11_) and ternary solid sulfide electrolyte (composed of Li_2_S, P_2_S_5_, MS_2_, M = Si, Ge, Sn, such as Li_10_GeP_2_S_12_). According crystallinity difference, the two kinds of SSEs can be further divided into glass, glass ceramic, and ceramic form solid electrolyte, which exhibit different performance in terms of ionic conductivity, chemical stability, and contact with solid electrode.

**Figure 5:**
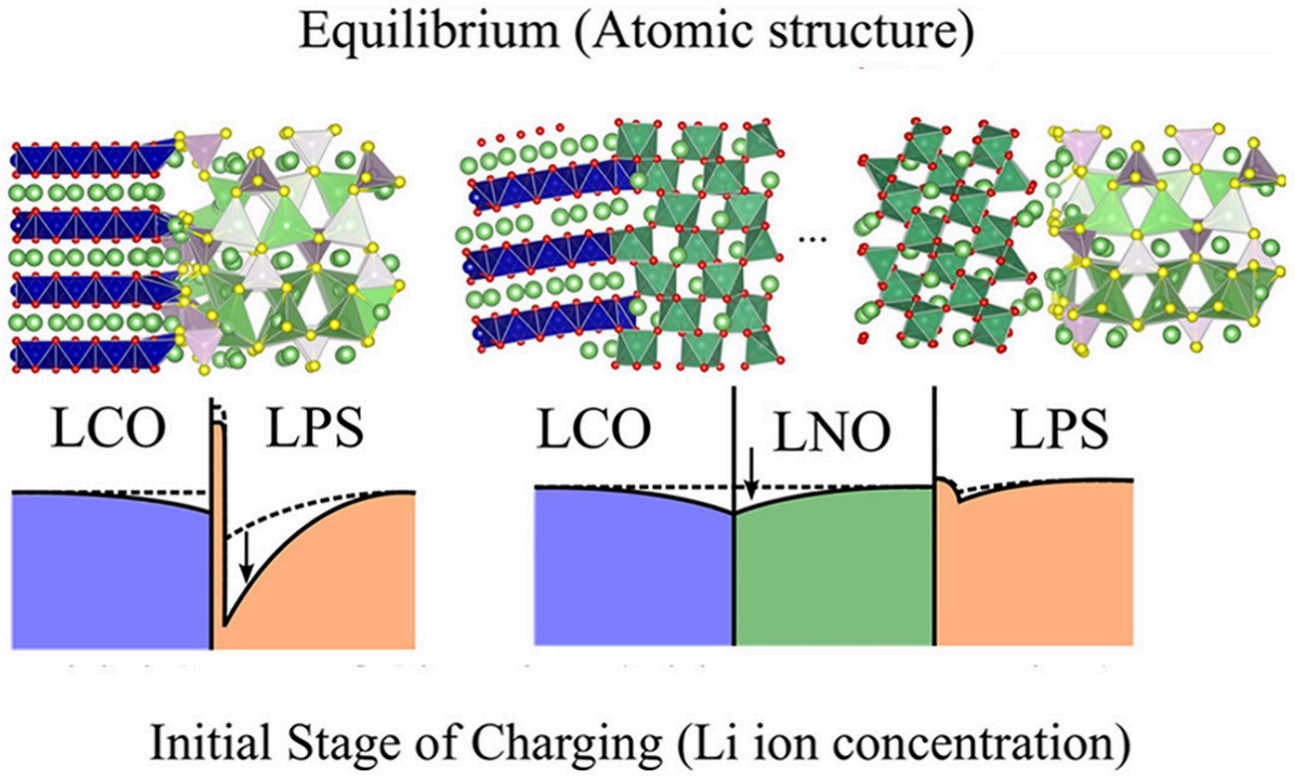
Comparison of atomic structures (at equilibrium state) and Li-ion concentration (at initial stage of charging) at the LiCoO_2_/Li_3_PS_4_ interfaces without (left) and with (right) LNbO_3_ buffer layer. [Reprinted with permission from (Haruyama et al., 2014). Copyright (2014) American Chemical Society].

Owing to Li^+^ chemical potential difference between oxide cathode and solid sulfide electrolyte, Li^+^ may migrate from electrolyte to cathode, resulting in SCL at both sides. Due to the mixed ionic and electronic conductivity in oxide cathode, Li^+^ gradient concentration can be compensated at cathode side. However, SCL will remain at electrolyte side due to the single ionic conductivity of electrolyte. The resulting SCL can well-impede Li^+^ transport and induce high polarization. SCL was firstly proposed by Wagner (1972) and extensively investigated on conduction type and conductivity change of composite materials, polycrystalline and heterojunctions (Liang, 1973; Maier, 1995; Bhattacharyya and Maier, 2004). With theoretically calculation, Haruyama et al. (2014) elucidated the characteristics of SCL between LiCoO_2_ and β-Li_3_PS_4_, the effect of LiNbO_3_ buffer layer interposition was also clarified. DFT calculation further revealed Li^+^-preferred adsorption at oxygen bridge sites, e.g., CoO_6_, and on Li layer, which may be the origin of deformed interface and SCL. Li chemical potentials based on vacancy formation energy indicate that the subsurface Li in sulfide electrolyte may transfer under electric field, suggesting that SCL grows immediately at the beginning of charging (Figure 5). Since the attractive sites on LiCoO_2_ surface disappear with insulating LiNbO_3_ layers attachment, the SCL at this interface is significantly suppressed. This result consistently explained SCL at atomic-scale and clearly indicated the effect of buffer layers. To eliminate SCL at the oxide cathode/SSE interface, oxide layer with high Li^+^ conductivity, and chemical stability (mostly LiNbO_3_) is always introduced at interface and combined with other modification techniques (Kitaura et al., 2011; Haruyama et al., 2017; Koerver et al., 2017).

**Figure 6:**
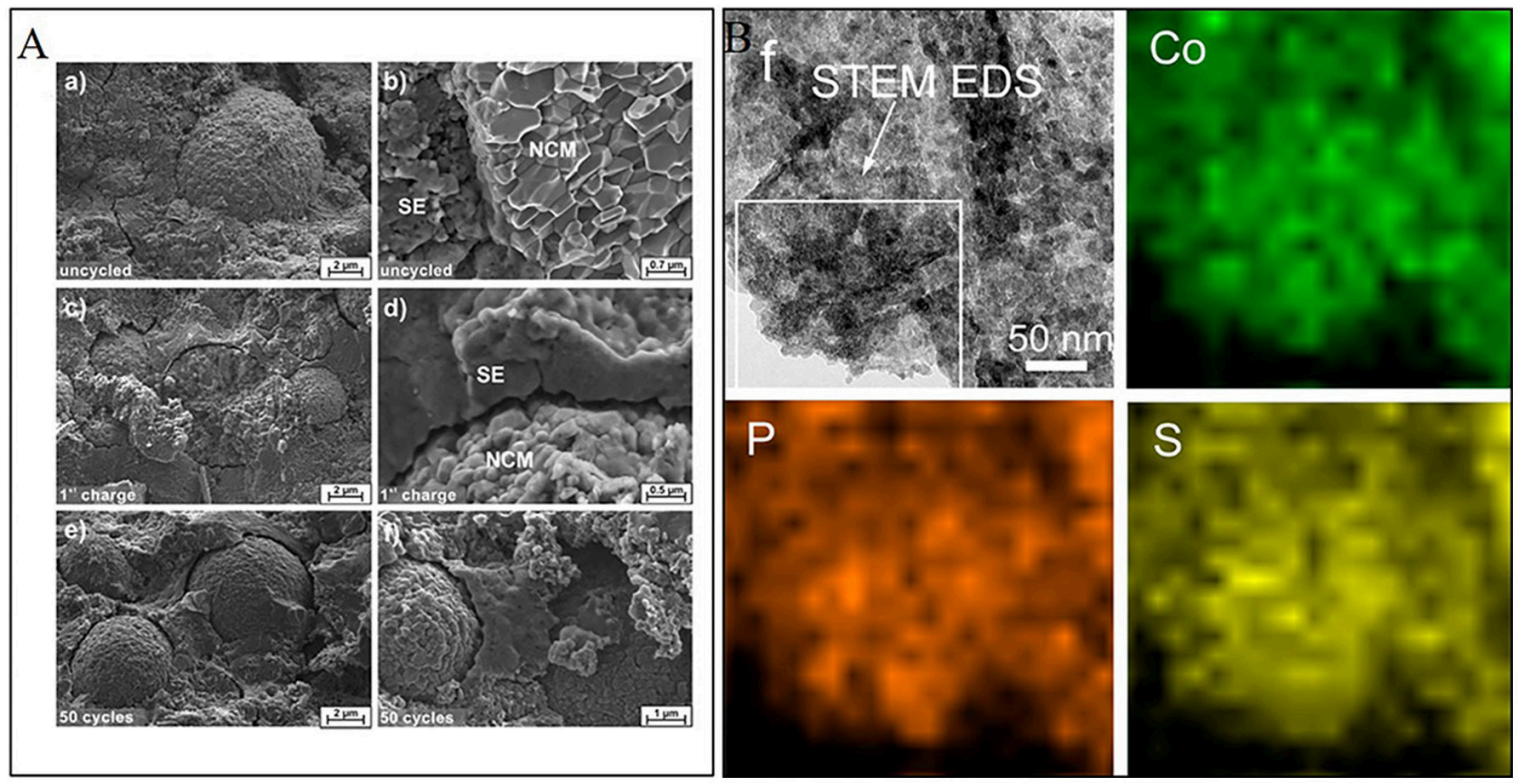
**(A)** Scanning electron micrographs of the cathode composite of NCM811 and β-Li_3_PS_4_: (a,b) before cycled; (c,d) after single charging to 4.3 V vs. Li/Li^+^ at 0.1 C; (e,f) after 50 full battery cycles in the discharged state. [Reprinted with permission from (Koerver et al., 2017). Copyright (2017) American Chemical Society]. **(B)** STEM EDS elemental mapping images of cobalt sulfide-Li_7_P_3_S_11_ nanocomposites. [Reprinted with permission from (Yao et al., 2016). Copyright (2016) American Chemical Society].

Even though solid sulfide electrolyte has moderate physical deformability, electrochemically driven mechanical failure also contributes to interfacial resistance increase and capacity fading. Koerver et al. evaluated the interfacial behavior for solid state lithium battery using nickel-rich NCM-811 cathode and β-Li_3_PS_4_ solid electrolyte (Koerver et al., 2017). Results suggest that the majority of passivating layer is developed during the first charge and present slow growth upon further cycling. It was further found that electrode-electrolyte contact lose occurs in first charging due to electrochemical contraction. The mechanical failure even deteriorates in the following cycles (Figure 6A) and lead to high polarization and capacity decay. In order to achieve and sustain intimate interface contact, different methods, and strategies have been developed and investigated. Sticking the supercooled liquid state of electrolyte on active material particles combined with a hot press was used to achieve an intimate electrode-electrolyte interface (Kitaura et al., 2011). In contrast to the interface formed by RT pressing, hot pressing at around T_g_ may well-obtain intimate contact along with an interfacial layer between LiCoO_2_ and 80Li_2_S·20P_2_S_5_ glass electrolyte. LiNbO_3_ coating layer can be further introduced to suppress the reaction of LiCoO_2_ with the 80Li_2_S·20P_2_S_5_ glass solid electrolyte. Yao et al. (2016) reported a general interfacial architecture, i.e., Li_7_P_3_S_11_ electrolyte particles (around 10 nm) anchored on cobalt sulfide nanosheets, by *in-situ* liquid-phase approach. The STEM-EDS elemental mapping of an individual nanocomposite in Figure 6B confirms that the cobalt sulfide–Li_7_P_3_S_11_ nanocomposites are homogeneously distributed throughout the nanosheets and have an intimate contact. The obtained intimate contact contributed to an excellent rate capability and cycling stability. Similar intimate contacts could be achieved by sulfide electrolyte coating onto active materials to form a favorable interface (Ito et al., 2017). By Mixing LiCoO_2_ particles with different grain sizes during the electrolyte coating process, higher packing density pellets with less voids were obtained both before and after cycling which ensured fine networks of ionically conductive pathways. Moreover, Oh et al. (2016) discovered continuous LGPS decomposition at LGPS/acetylene black (AB) interface above 4.5 V. The decomposition layer could also isolate the delithiated LixNi_0.5_Mn_1.5_O_4_ (x~0) from Li^+^ and/or electron conduction channels in cathode composite, resulting in contact loss, and severe capacity fading upon cycling. The research demonstrates that suitable conductive additive and sulfide solid electrolyte are crucial to overcome the poor cycle performance of high-voltage solid state lithium batteries. Yoon et al. (2018) also investigated the interface between Li_10_GeP_2_S_12_ and diverse carbon conductive agents in solid state lithium batteries and confirmed the solid electrolyte decomposition and surface degradation during cycle.

### Solid Composite Electrolyte Design and Interface Optimization

Solid composite electrolyte is a subset of polymer electrolytes by dispersing electrochemically inert fillers, such as Al_2_O_3_ and TiO_2_ nanoparticles or inorganic solid electrolyte into polymer electrolyte. (Weston and Steele, 1982; Croce et al., 1998, 1999) These composite electrolytes have excellent mechanical stability (due to the ceramic fillers into polymer network) and high ionic conductivity (promoted by the high surface area of the dispersed fillers). Due to the absence of liquid components and interfacial stabilizing action from dispersed fillers, composite electrolyte offers wide electrochemical stability window (Croce et al., 1999). With advantages such as high ionic conductivity, wide electrochemical stability window, favorable interface mechanical properties, composite electrolytes have attracted extensive attention.

**Figure 7:**
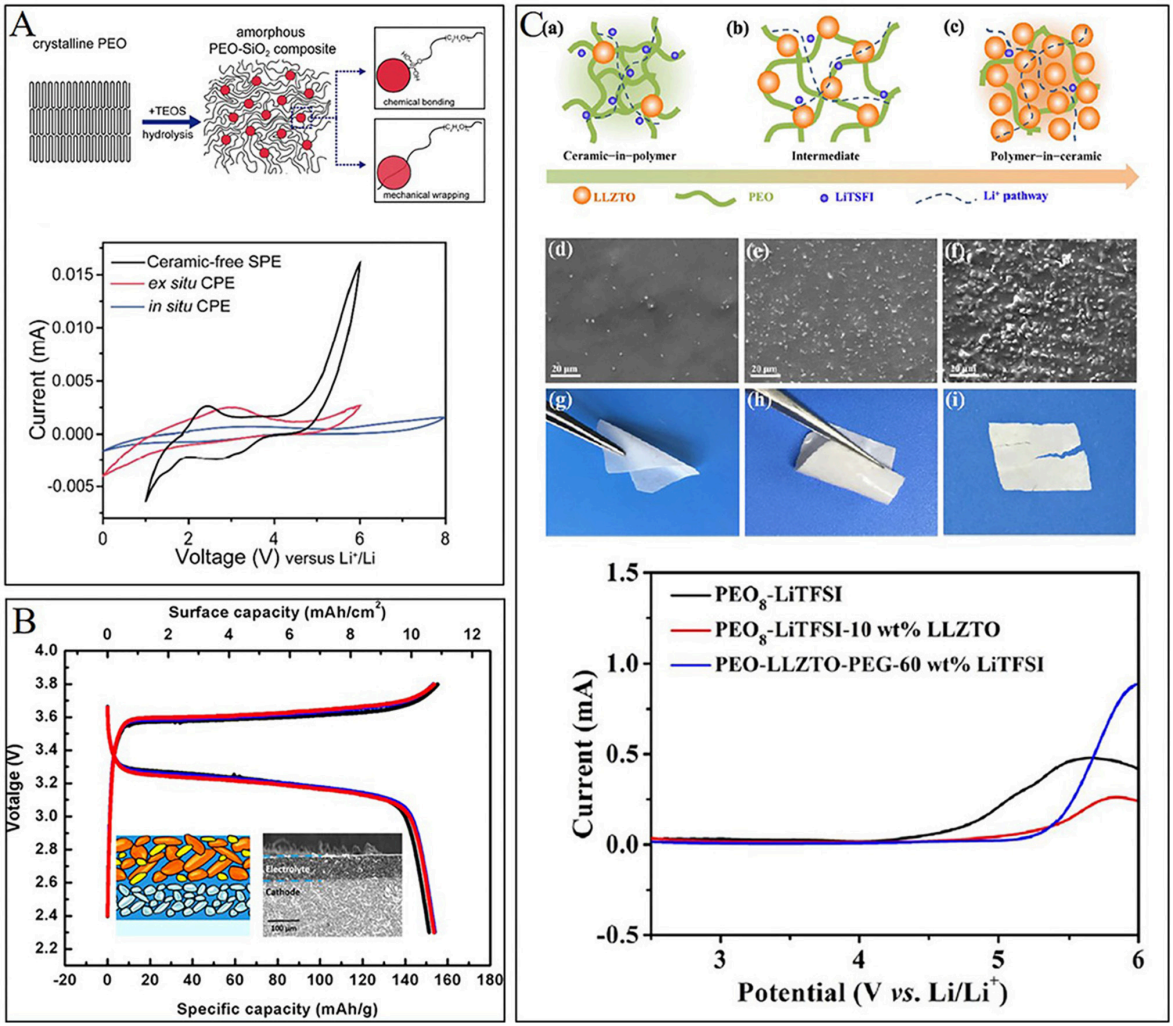
**(A)** Schematic figures showing the procedure of *in situ* hydrolysis and interaction mechanisms among PEO chains and MUSiO_2_ (up) and the electrochemical stability windows curves of three kinds of solid electrolyte. [Reprinted with permission from (Lin et al., 2016). Copyright (2016) American Chemical Society]. **(B)** The sketch map SEM image of the interface between Al-LLZTO/PEO composite cathode containing 15 wt% polymer and the composite electrolyte. The curves refer to first three galvanostatic charge and discharge curves. [Reprinted with permission from (Chen et al., 2018a). Copyright (2018) American Chemical Society]. **(C)** Schematic illustration for PEO-LLZTO solid composite electrolyte: (a) “ceramic-in-polymer”; (b) “intermediate”; (c) “polymer-in-ceramic”; the typical surface morphologies and flexibility of composite electrolyte (1-x) wt%[PEO_8_-LiTFSI]-x wt% LLZTO: (d,g) 10 wt%; (e,h) 50 wt%; (f,i) 80 wt%; the liner sweep voltammograms for different compositional solid composite electrolytes at 55°C with a scan rate of 1 mV s^−1^. [Reprinted with permission from (Chen et al., 2017). Copyright (2017) American Chemical Society].

The inorganic fillers in solid composite electrolytes could be oxides without Li^+^ conducting ability, such as Al_2_O_3_, TiO_2_, SiO_2_, etc. (Lin et al., 2016; Pal and Ghosh, 2018) and other solid electrolytes, such as LLZO, LAGP, LGPS, etc. (Zhao et al., 2016; Chen et al., 2017, 2018a,b; Zhai et al., 2017). In 2016, Lin et al. introduced a novel *in-situ* synthesis of a SiO_2_ filler inside PEO polymer. Much stronger chemical/mechanical interactions between SiO_2_ nanospheres and PEO chains can be obtained, which significantly suppresses PEO crystallization and facilitates polymer segmental motion for Li^+^ conducting (Lin et al., 2016). Two possible interaction mechanisms are shown in Figure 7A, including chemical bonding between PEO chains and hydroxyl groups on MUSiO_2_ and mechanical wrapping of PEO chains during MUSiO_2_ spheres growth. At the same time, electrochemical stability window can be largely extended up to 5.5 V, much wider than *ex-situ* CPE and ceramic-free CPE (Figure 7A). The improvement of electrochemical stability indicates that the adsorption effect on anion is much stronger in *in-situ* CPE, which suppresses anodic decomposition at high potential (Park et al., 2003).

Garnet type LLZO electrolyte, has been widely studied as kind of filler in PEO matrix (Zheng et al., 2016; Chen et al., 2017, 2018a; Zhang et al., 2017a). Recently, Chen et al. (2018a) proposed a synergistic-composite approach to fabricate flexible solid state lithium batteries using PEO-based composite cathode layers (filled with LiFePO_4_ particles) and composite electrolyte layers (filled with Al-LLZTO particles) which exhibits a wide electrochemical stability window ~6 V, much wider than pure PEO. The all-composite approach is favorable for improving both mesoscopic and microscopic interfaces (Figure 7B) inside solid state lithium batteries and may provide a new toolbox for solid state lithium batteries design and fabrication. The interface between composite cathode and composite electrolyte layers may keep its structural integrity albeit the large volumetric change during cycling (Chen et al., 2017). Since the synergetic-composite electrolyte combines the virtues of two components, compositing stands a chance in building favorable interfaces, and further realizing high energy density solid state lithium batteries. Chen et al. (2018a) prepared composite ceramic/polymer solid electrolyte containing garnet Li_6.4_La_3_Zr_1.4_Ta_0.6_O_12_ (LLZTO), PEO, and Lithium bis(trifluoromethanesulfonyl)imide (LiTFSI). The composite solid electrolyte possesses high self-standing and flexibility, which exhibits electrochemical stability window up to 5.0 V vs. Li/Li^+^. The assembled solid-state LiFePO_4_|Li batteries with electrolytes from “ceramic-in-polymer” to “polymer-in-ceramic” exhibit excellent cycling stability and wide electrochemical stability window (more than 5.0V vs. Li^+^/Li). The “ceramic-in-polymer” electrolyte exhibits a greater flexibility (Figure 7C) and lower cost, while “polymer-in-ceramic” electrolyte shows higher mechanical strength and safety but brittler for bend cracks formation. Hence, by varying the composition of composite electrolyte, different properties will be obtained for special applications. Apart from LLZO, other oxide solid electrolyte, such as LATP and LAGP are also widely combined with PEO in order for better composite solid electrolyte (Wang et al., 2017b; Zhai et al., 2017).

Compared with TM oxide particles, solid sulfide electrolyte, such as LGPS and Li_3_PS_4_, exhibits fast ionic conductivity (Han et al., 2011; Haruyama et al., 2014; Kato et al., 2016). As a result, solid sulfide electrolyte incorporate into PEO matrix can provide excellent Li^+^ conducting channel. Zhao et al. fabricated SPE membranes comprised of LGPS and PEO matrix. The optimal composite membrane exhibits high ionic conductivity ~1.21 × 10^−3^ S cm^−1^ at 80°C and wide electrochemical window of 0–5.7 V (Zhao et al., 2016). Instead of simply mixing ceramic particles with polymers, the same group prepared PEO/Li_3_PS_4_ hybrid polymer electrolyte via new *in-situ* approach (Chen et al., 2018b). The optimal electrolyte of PEO-2% vol Li_3_PS_4_ presents the highest Li^+^ conductivity and widest electrochemical window. In anodic process, *in-situ* prepared electrolyte shows no anodic current until 5.1 V, while corresponding voltage for mechanical-mixed electrolyte and PEO are 4.9 V and 4.6 V, respectively, as reported in their article. The differences in ionic conductivity and stability may originate from more homogeneous dispersion of fillers in PEO by *in-situ* preparation than by mechanical-mixing.

According to the mechanical properties including flexibility, deformability, and strength of the aforementioned four kinds of electrolytes, the different interface performance and modifications at cathode side are summarized in Table 1.

**Table 1:** Interfacial challenges exist in cathode-solid electrolyte systems according to the different characteristics of the four types of solid electrolytes and the corresponding solutions, recent advances and limitations still exist.

**Interfaces**	**Interfacial mechanical performance**	**Interfacial chemical stability**	**Solutions and advances**	**Limitations still exist**
Cathode-Solid polymer electrolyte interface	Excellent elasticity and deformability promote favorable interface contact Poor strength cannot block Li dendrites	PEO-based SPE is not stable above 4.0 V (Croce et al., 2001)	(a) Optimization of Li salts (Zhang et al., 2011; Ma et al., 2016b) (b) Polymer matrix modification such as copolymerization, branching and crosslinking (Tong et al., 2014; Porcarelli et al., 2016) (c) Gel/ plasticized polymer electrolyte (Manuel Stephan, 2006; Zewde et al., 2018) (d) Cathode coating (Ma et al., 2017; Yang et al., 2018)	(a) Performance matched with high-voltage cathode such as LiCoO_2_ and Li_2_MnO_4_ is still poor (b) The weaknesses of liquid electrolyte still exist in gel system, such as flammable property (c) Short-circuit concerns
Cathode-solid oxide electrolyte interface	High strength properties can partially block dendrite Poor flexibility lead to a poor solid-solid contact Dendrite can grow along grain boundaries	Stable up to 6V (Li et al., 2015; Thangadurai et al., 2015) High-temperature handling (>500°C) to may lead to elements interdiffusion and form transition layer	(a) Surface coating (Han et al., 2017) (b) Co-sintering (Wakayama et al., 2016) (c) In-situ synthesized electrolyte layer (Yoshima et al., 2016; Kazyak et al., 2017) (d) Interface softening (Seino et al., 2011; Sakuda et al., 2012; Liu et al., 2016) (e) Interface buffer layers (Kato et al., 2014; Park et al., 2016) (f) Amorphous cathode (Matsuyama et al., 2016; Nagao et al., 2017)	(a) Even intimate contact could be achieved at pristine state, contact loss will happen upon cycling during to the rigid ceramic nature (b) High interface resistance prohibits thicker cathode layer and high capacity battery as a result
Cathode-solid sulfide electrolyte interface	Reasonable strength and decent deformability Poor elasticity lead to contact loss upon periodic cathode expanding and shrinking	High Li chemical potential leads to a space charge layer when matched with oxide cathodes Electrochemically unstable when contacted with high-voltage cathode	(a) Interface buffer layer to mitigate SCL (Haruyama et al., 2014; Koerver et al., 2017) (b) Hot press, *in-situ* synthesis and sulfide electrolyte coating onto active materials to obtain intimate contact (Kitaura et al., 2011; Yao et al., 2016; Ito et al., 2017)	Contact loss upon cycling is still an unsolved problem which makes external pressure necessary
Cathode /solid composite electrolyte interface	Combine the virtues of both polymer and ceramic with both reasonable strength and flexibility, promising to obtain favorable contact	By adding inorganic fillers in PEO based solid electrolyte, the anti-oxidation property at high voltage is still under discussion even various studies reported high electrochemical window	By regulating the composition of composite electrolyte, solid electrolytes with different performance will be obtained to adapt to different requirements	Drawbacks exist in single solid electrolyte system such as poor stability of SPE, SCL in solid sulfide electrolyte and poor flexibility of solid oxide may still exist when these components contact cathode A novel solid electrolyte with high ionic conductivity, chemical stability Compatibility with cathode is still a long way to go

## Advanced Solid-Solid Interface Characterization Techniques

As discussed above, the interface behaviors play an important part in determining the final solid state lithium battery parameters and performances, including internal resistance, kinetic response, and cycle stability etc. However, the buried solid-solid interfaces in solid state lithium batteries are extremely difficult to investigate directly, and present knowledge on interfacial reactions and interfacial kinetics is still deficient. As a result, it is increasingly important and urgent to develop novel characterization techniques for more detailed understanding into the interface behavior (Hu et al., 2006). Note that the solid electrolyte-cathode interface involves several aspects correlated with each other, including lattice structure, electronic band structure, and chemical/electrochemical stability. The dynamic Li shuttle back and force across the interface further makes the interface behavior more complicated within time and voltage domain. Hence, advanced characterization techniques with *in-situ* and atomic-scale resolution are strongly necessary to gain more insights into the complex interface processes (Zheng et al., 2014; Lin et al., 2017). So far, diverse advanced characterizations have been utilized by different research groups worldwide and significant information have been obtained. These research works provide valuable perspective of interface performance and have profound guiding significance for designing more favorable interfaces in superior solid state lithium batteries.

Since a significant reason for interfacial instability is the abrupt change of electric potential across the cathode-electrolyte interface, dynamic observation of the potential profiles would help identify sources of typically large interfacial resistance. Ogumi' group contributed a lot in studying the potential distribution and interface stability mechanisms in solid state lithium batteries (Yamamoto et al., 2010, 2012; Okumura et al., 2011). With this objective, EH (quantitative electron holography) combined with EELS (electron energy loss spectroscopy) was used to directly observe the potential distribution at the LiCoO_2_/Li_1+x+y_A_y_Ti_2−y_Si_x_P_3−x_O_12_ interface (Yamamoto et al., 2010). Results showed the Li^+^ and electron typical distribution of the measured potential near the cathode-electrolyte interface during charging and the origin of the shift of the electronic band structures. This research identified the sources of reaction resistance and kinetic factors in solid state lithium battery. EH also clearly show how the metallic lithium is formed inside the solid electrolyte during the initial charging process of the solid state lithium battery (Yamamoto et al., 2012). Results showed that the smooth potential distribution at the electrode/solid electrolyte interface leads to the low interfacial resistance.

**Figure 8:**
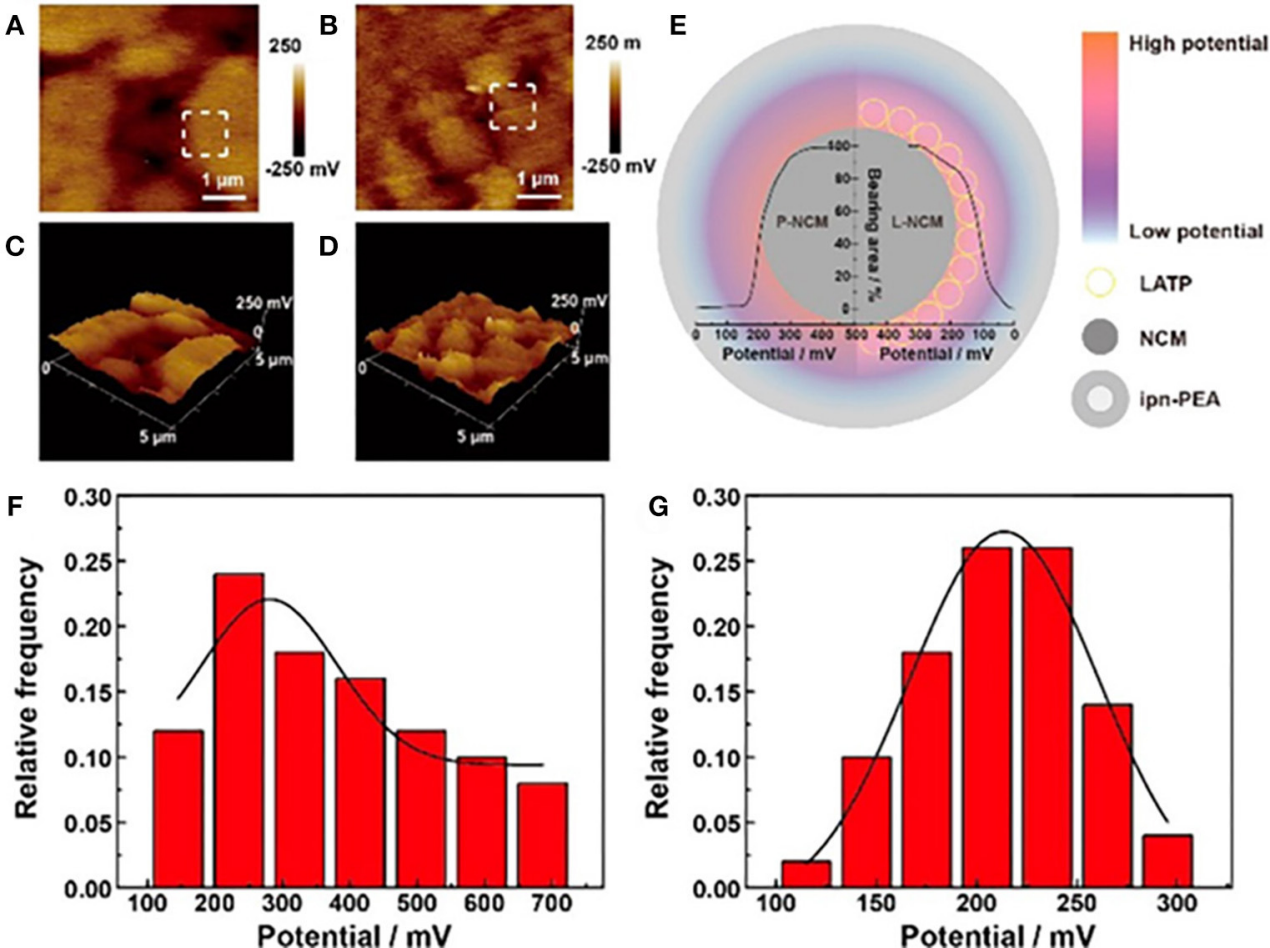
AFM interfacial potential images and the corresponding 3D images of **(A,C)** pristine-NCM and **(B,D)** LATP coated-NCM. **(E)** Schematic diagram with bearing analysis and potential distribution of the two cathodes. The Gauss statistic distribution histograms of interfacial potential for **(F)** pristine-NCM and **(G)** LATP coated-NCM. [Reprinted with permission from (Liang et al., 2018). Copyright (2018) American Chemical Society].

With the unique sensitivity and operability in SCL detection, AFM was performed to better understand the potential distribution at the cross section of particles (Liang et al., 2018). By introducing LATP coating at cathode surface, the LiNi_0.6_Co_0.2_Mn_0.2_O_2_/ poly(ether-acrylate) (ipn-PEA) interface realized a mitigated polarization and excellent kinetic performance. The significantly improved interface dynamics is attributed to a weakened SCL or a gradual slope of potential formed at the interface, verified by AFM interfacial potential measurements, which relieves polarization, alleviates side reactions, and enhances cycling stability and dynamic properties (Figure 8).

Apart from interfacial potential research techniques, advanced characterizations adopted to investigate structural and chemical stability of the interface also promote the mechanism understanding of the interface behavior. *In/ex situ* characterization techniques including spectroscopy, microscopy, and diffractometry are widely used to monitor the structural evolution and chemical reactions at the interface.

**Figure 9:**
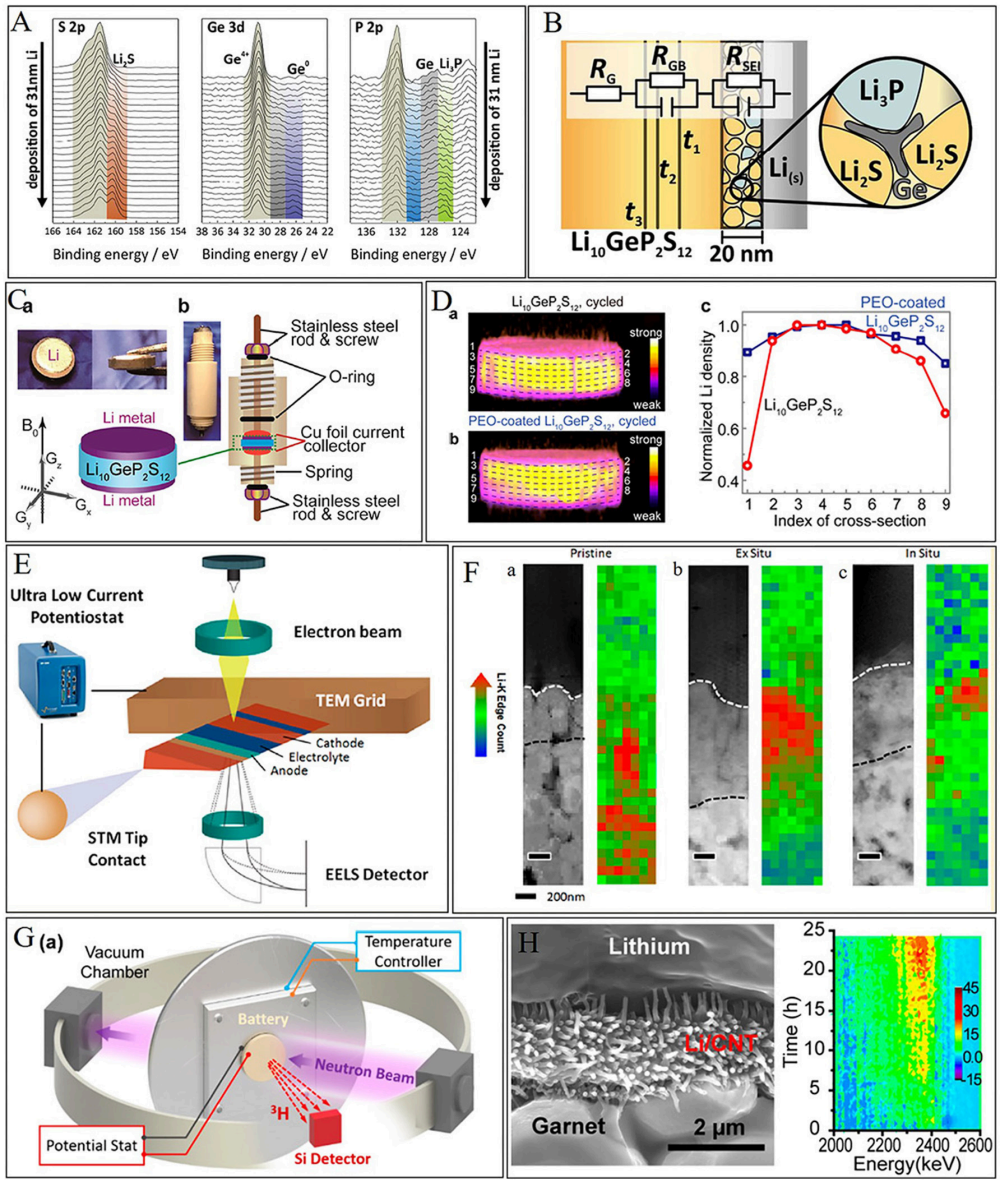
*In situ* characterization techniques for solid-solid interface stability. **(A)** *in situ* XPS recorded during deposition of Li metal on LGPS. With increasing Li deposition time, LGPS decomposes. **(B)** Schematic of the interphase formation at the Li/LGPS interface according to the XPS result. [Reprinted with permission from (Wenzel et al., 2016). Copyright (2016) American Chemical Society]. **(C)** Pictures and schematic of a cylindrical cell for MRI. **(D)** Li density profiles at different depths of electrochemically cycled LGPS pellets. [Reprinted with permission from (Chien et al., 2018). Copyright (2018) American Chemical Society]. **(E)** Schematic of *in situ* TEM biasing of nanobattery. **(F)** STEM image and EELS characterization. (a–c) HAADF image of the nanobattery stack along with Li K-edge concentration mapping of (a) pristine, (b) *ex situ*, and (c) *in situ* samples with scale bar represents 200 nm. [Reprinted with permission from Wang et al. (2016b). Copyright (2016) American Chemical Society]. **(G)** Schematic of the NDP system. **(H)** 2D projection of the NDP spectra collected at 5 min intervals during cycling. [Reprinted with permission from (Wang et al., 2017a). Copyright (2017) American Chemical Society].

Spectroscopy including XAS (X-ray absorption spectroscopy), XPS (X-ray photoelectron spectroscopy), NMR (Nuclear Magnetic Resonance Imaging), etc. in an *in-situ* mode with high spatial resolution play important role in the solid-solid interface understanding. Okumura et al. (2011) developed a depth-resolved XAS to directly observe the chemical state and local structure at the LiCoO_2_/LATP interface with/without NbO_2_ modification layer. XAS results revealed that the introduction of NbO_2_ layer is effective for restricting the large Co–O bond change at the interface during delithiation. As a result, the charge transfer process is smoother owing to a relieved interface stress. Wenzel et al. developed *in-situ* XPS by using the internal argon ion gun of the instrument which was adopted to sputter a metallic target (Wenzel et al., 2015). The chemical stability of the LLTO/metallic lithium interface was investigated. The same *in-situ* XPS method was adopted by the same group to investigate the interface between lithium metal and Li_10_GeP_2_S_12_. XPS recorded the decomposition products which revealed the formation of lithium sulfide, lithium phosphide, and germanium-lithium alloy/germanium metal (Figures 9A,B) (Wenzel et al., 2016). *In situ* NMR is also widely used to study the lithium distribution in solid lithium batteries (Bhattacharyya et al., 2010; Nakayama et al., 2010; Wang et al., 2014; Romanenko et al., 2016; Chien et al., 2018). Very recently, three-dimensional ^7^Li magnetic resonance imaging (MRI) is employed to examine Li distribution homogeneity in solid electrolyte Li_10_GeP_2_S_12_ within symmetric Li/Li_10_GeP_2_S_12_/ Li batteries by Chien et al. (2018) (Figure 9C). The three-dimensional Li distribution revealed that the significant Li loss at interfaces is mitigated via PEO coating (Figure 9D) (Chien et al., 2018). This study demonstrates a powerful tool for non-invasively monitoring the Li distribution at the interfaces and in the bulk of solid state lithium batteries as well as a convenient strategy for improving interfacial stability. As mentioned in chapter 3, TOF-SIMS can also be used to study the interface element distribution around the interface in solid state lithium batteries (Park et al., 2016).

Microscopy [e.g., TEM (transmission electron microscope), STEM (scanning transmission electron microscopy), etc.] techniques are powerful tools to investigate the structural and chemical stability of solid electrode/solid electrolyte interface. Wang et al. (2016b) used *in situ* STEM-EELS with high spatial resolution observed the interfacial phenomena of LiCoO_2_/LiPON with a nanoscale resolution (Figure 9E). An unexpected structurally disordered interfacial layer was discovered without cycling. The interfacial layer accumulates lithium and evolves to rock salt CoO after cycling along with Li_2_O and Li_2_O_2_ formation (Figure 9F) (Wang et al., 2016b). Rapid capacity decay or even cathode inactivation will happen along with the thickening of this layer. *In situ* STEM was also introduced to study the interface stability of lithium metal/solid electrolyte (Ma et al., 2016a). Gong et al. (2017) designed an *in situ* atomic-scale TEM observation of electrochemical delithiation induced structure evolution of LiCoO_2_ cathode in solid state lithium batteries, which provides atomic-scale structure information for designing better solid state lithium batteries.

*In situ* diffractometry including XRD (X-ray diffraction) and ND (neutron diffraction) were also widely used to monitor the structural change upon cycling in solid state lithium batteries (Shin et al., 2014; Wang et al., 2017a; Hu et al., 2018). Wang et al. developed an *in situ* ND technique to monitor the Li distribution and transport in garnet-based solid-state cells during cycling (Figures 9G,H) (Wang et al., 2017a). When Li is deposited outside the reversible layer, it becomes “dead lithium”. A 3D mixed electron–ion conductive framework is preferred as a Li metal host to increase the contact area, shorten the Li diffusion distance, and overcome the anticipated volume change.

## Conclusion and Perspectives

This review provides a brief survey of recent research and development with respect to cathode–solid electrolyte interfaces in solid-state lithium batteries. We summarized the basic electrochemistry and principle at cathode-solid electrolyte interface, fundamental factors inducing interface challenges, and research progresses on building better interfaces. The interface issues in solid organic electrolytes, solid inorganic electrolytes, and solid composite electrolytes were reviewed and corresponding solutions are summarized on the basis of intrinsic characteristics of different solid electrolyte.

The interface degradation in solid state lithium battery may stem from the chemical/electrochemical stability and mechanical stability. Poor chemical/electrochemical stability between cathode and electrolyte may cause electrolytes decomposition or elements interdiffusion and transition region formation. The fundamental mechanism of interfacial chemical instability lies in the distribution of ionic electrochemical potential and inner electric potential gradient. Electronic and ionic conductivity of the transition region fundamentally determine whether a stable interface will form or not. An ionic conducting and electronic insulating transition layer will prevent further oxidation/reduction of electrolytes and SCL growth. While a mixed ionic and electronic conducting layer will result in continuous reactions and transition region growth.

To improve the chemical stability at cathode/SPE interface, different strategies can be adopted for different types of solid electrolytes. Modification of PEO matrix and lithium salts, adding proper plasticizer, cathode coating, and compositing with inorganic fillers are favorable for SPE and have made great progress. Solid oxide electrolytes are fairly stable with cathode compared with other solid electrolytes. While interdiffusion will take place along with high temperature dealing during *in-situ* synthesis, co-sintering, and deposition. Proper modification layer is needed to guarantee both chemical stability and intimate contact. Solid sulfide electrolytes, which suffer from SCL problem and decomposition reactions, should introduce other surface modifying method on the basis of introducing a proper buffer layer to eliminate SCL.

In addition to the chemical instability, it's difficult for inorganic solid electrolytes especially solid oxide electrolyte to maintain intimate contact with cathode due to the rigid ceramic nature. To achieve good mechanical contact, strategies such as surface coating, co-sintering, *in-situ* synthesis of electrolyte layer, interface softening, interface buffer layers were employed. By interface modification, the above issues are mitigated to a large degree, while a novel solid electrolyte with high ionic conductivity, chemical stability, compatibility with both cathode, and anode is still a long way to go.

In order to gain more insights into interface behavior, advanced characterization techniques are necessary, particularly with time, and atomic-scale resolution. Recent investigations and progress obtained from diverse advanced characterization techniques are summarized here. Note that interface phenomena involve multiscale and multidimensional properties change, the combination of diverse technologies may help provide comprehensive understanding. Electron holography and internal potential from AFM help uncover the electric potential profiles across interface. Spectroscopy including XAS, XPS, and NMR directly probes the chemical state and local structure. Microscopy including TEM and STEM provide atomic arrangement at interface structure. While XRD and ND can well-monitor structure evolution upon battery cycle. MRI and TOF-SIMS may further build up 3-dimensional distribution of particular elements. Despite valuable information obtained from these tools, here we highlight that the wider cooperation between diverse techniques will provide stronger support to the final clarification of interface related phenomena.

According to detailed analysis of interface issues and solutions for different systems, we further conclude that to realize a favorable interface, a hybrid solution should be employed to achieve both mechanical and chemical/electrochemical stability. It's hard for one kind of solid electrolyte to combine both excellent elasticity to achieve intimate contact and favorable strength to resist lithium dendrite formation. On the one hand, an elastic and transformable electrolyte which could shrink and expand in pace with cathode is essential for solid state lithium batteries, such as SPE and special solid sulfide electrolyte. Note that even deformable sulfide electrolyte could not keep contact with electrode particles upon cycling, therefore other strategies such as *in-situ* synthesis and surface coating may serve as proper ways to modify solid sulfide electrolytes. In order to obtain pristine intimate contact before cycling, strategies such as flash-burning, interface sintering, deposition method, and *in situ* polymerization are necessary. Meanwhile, to relieve the strain and stress resulted from the shrinkage and expansion of cathode material, nano-sized solid electrolyte and electrode may serve as a good choice. On the other hand, deficiency in strength and toughness of sulfides and polymers calls for toughening or compositing with other solid electrolytes.

Chemical/electrochemical stability are equally important, theoretical calculations, and some experimental results both revealed that the actual stability windows of solid electrolytes are not wide as expected. A stable interface layer with high Li^+^ conductivity and low electrical conductivity is expected. Li-compounds like LiF, Li_2_S, Li_2_O, Li_3_N, and LiNbO_3_ are favorable interface components, while electronic conducting constituents such as metal sulfides (e.g., CoS) and Li-Metal alloys (e.g., Li-Ge alloy) should be avoided. To combine both mechanical and chemical/electrochemical demands of solid electrolyte, developing SPE with high strength and wide electrochemical window is necessary. Compositing is a promising method to utilize synergy effects among various electrolytes, but mitigating the disadvantages of each component need further study. Gel softening interface is also a promising way to achieve intimate contact at electrode-electrolyte interface. But flammable liquid components with narrow electrochemical stability window should be avoided.

According to various requirements, creating an ideal cathode-solid electrolyte interface requires a combination of various factors and methods. By building favorable electrode-electrolyte interface, solid-state batteries with higher safety performance, longer cycle life, and higher energy density are predictable.

## Author Contributions

All authors listed have made a substantial, direct and intellectual contribution to the work, and approved it for publication.

### Conflict of Interest Statement

The authors declare that the research was conducted in the absence of any commercial or financial relationships that could be construed as a potential conflict of interest.
